# Physician Modified Endograft for Ruptured Dissecting Aortic Arch Aneurysm

**DOI:** 10.1177/15385744241276599

**Published:** 2024-08-20

**Authors:** Antonio Solano, Melissa R. Keller, Jesus Porras Colon, Rhusheet Patel, Carlos H. Timaran, Melissa L. Kirkwood, M. Shadman Baig

**Affiliations:** 1 Division of Vascular and Endovascular Surgery, Department of Surgery, 12334 University of Texas Southwestern Medical Center, Dallas, TX, USA

**Keywords:** aortic arch, aortic dissection, aortic rupture, physician-modified endograft, thoracic aortic aneurysm, thoracic endovascular aortic repair

## Abstract

**Background:**

Endovascular repair of thoracic aortic aneurysms (TAA) in elective settings has demonstrated successful clinical outcomes. However, life-threatening conditions such as rupture are more often managed with open surgical repair due to the high complexity of arch endovascular repair, lack of available off-the-shelf devices, and limited long-term data.

**Case Summary:**

A 49-year-old female with a recent history of prior ascending aortic repair for Type A_10_ aortic dissection presented with chest pain and dyspnea. Chest computed tomography angiogram (CTA) revealed acute bilateral pulmonary emboli and a 6.2 cm post dissection aneurysm of the posterior aortic arch with the dissection extending to the right iliac artery. She was treated with thrombolysis and subsequently became hemodynamically unstable. Repeat CTA revealed a massive left hemithorax with concern for aortic arch rupture. Given significant cardiorespiratory compromise and recent open repair, she was considered unfit for redo open repair. Thoracic endovascular aortic repair (TEVAR) with a physician-modified endograft (PMEG) was planned. An Alpha Zenith endograft was modified adding an internal branch for the innominate artery and a fenestration for the left common carotid artery. The left subclavian artery was occluded with a microvascular plug and coil embolization up to the level of the vertebral artery. TEVAR PMEG extension to the celiac artery was performed followed by deployment of a Zenith dissection stent to the aortic bifurcation. Completion angiogram demonstrated successful aneurysm exclusion and patency of target vessels.

**Conclusion:**

Endovascular treatment of ruptured TAA with PMEGs is feasible. This approach may be an alternative for unfit patients for open repair in emergent settings.

## Introduction

Thoracic aortic aneurysms (TAA) can be a life-threatening condition given the risk of rupture, with significant morbidity and mortality.^1^ TAA rupture incidence rates ascend to 5.0/100.000 inhabitants, with 50% risk for patients with large descending TAAs.^2-4^ Treatment ranges from close observation to endovascular or open repair based on etiology, clinical symptoms, size, growth rate and patient suitability. Intervention is recommended when rupture risk is higher than surgical complication risk.^5^ Natural history studies have demonstrated a high rate of rupture of unrepaired TAA.^4,6,7^ The Society for Vascular Surgery (SVS) guidelines recommend thoracic endovascular aortic repair (TEVAR) as the preferred approach for descending thoracic aortic (DTA) aneurysms given its reduced morbidity, length of stay, and short-term mortality in elective and emergent settings.^8^ However, TAAs involving the transverse aortic arch do not have readily available endovascular options and require open or hybrid repair with debranching of the supraaortic trunks followed by endograft placement into proximal healthy aorta. In patients presenting with rupture of a TAA involving the arch, a totally endovascular repair has the potential for rapid stabilization and avoidance of cardiopulmonary bypass. Herein, we present the case of a physician modified endograft (PMEG) for repair of a ruptured post dissection thoracic aneurysm involving the aortic arch. Verbal consent was obtained from the patient for publication of this case report.

## Case Report

**Figure 1.**
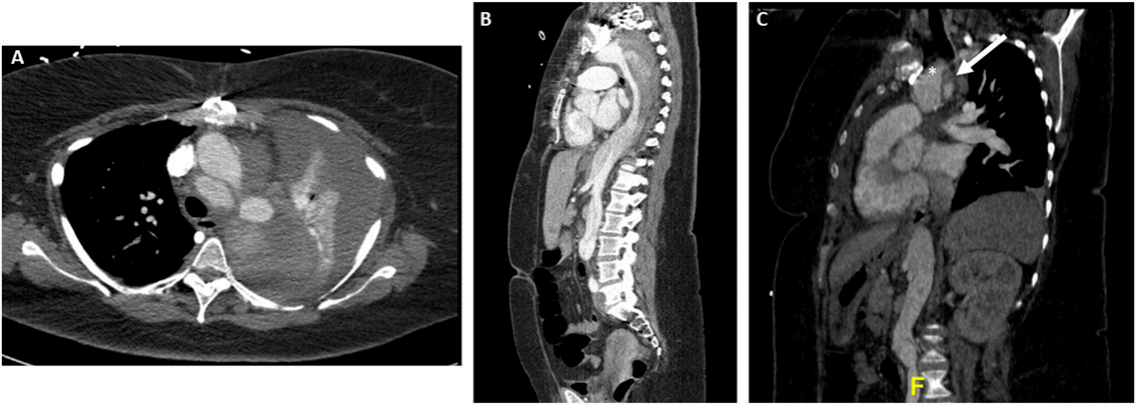
Axial (A) and sagittal (B) preoperative computed tomography angiogram (CTA) with massive left hemothorax and dissected aneurysm extending from the descending thoracic aorta to the right common iliac artery. (C) Close-up sagittal cut with demonstration of the dissection flap (arrow) and innominate artery origin (asterisk).

A 49-year-old female presented to an outside hospital (OSH) primary care physician visit with complaints of tachycardia and shortness of breath. Past medical history was significant for hypertension, dyslipidemia, paroxysmal atrial fibrillation, coronary artery disease treated with percutaneous transluminal angioplasty, and an ascending aortic repair for a type A_10_ dissection one month prior. The patient was transferred to the emergency department, and lower extremity venous ultrasound reported nonocclusive thrombus within the right common femoral vein at the level of the greater saphenous junction plus occlusive thrombus in the right calf veins and one of the paired left posterior tibial veins. Chest computed tomography angiogram (CTA) revealed bilateral segmental pulmonary embolism (PE) with a saddle embolus and a 6.2 cm post dissection aneurysm of the distal arch. Given these findings, she was admitted to the ICU with heparin infusion and subsequent thrombolysis with bilateral pulmonary artery EKOS catheter placement. The patient then experienced a significant drop in hemoglobin from 9.5 to 6.4 g/dL and a repeat chest CTA revealed proximal DTA rupture and massive left hemothorax. Urgent airlift transfer was requested for cardiac and vascular surgery consultation at our institution. Upon arrival, the patient had significant respiratory distress (55 respirations per minute with oxygen at 10 L/min with high flow nasal cannula), and tachycardia (130 beats per minute). STAT CTA for surgical planning was performed (Figure 1). A multidisciplinary discussion with cardiac surgery was held to discuss potential treatment options. Given the large left hemothorax, hypoxia, pulmonary embolism, and very recent (1 month post-op) ascending aortic repair, the patient was deemed a poor candidate for open repair. Ultimately, we elected to proceed with an arch branched PMEG TEVAR after a full discussion with the patient with regards to the risks of physician modification of standard endografts.

**Figure 2.**
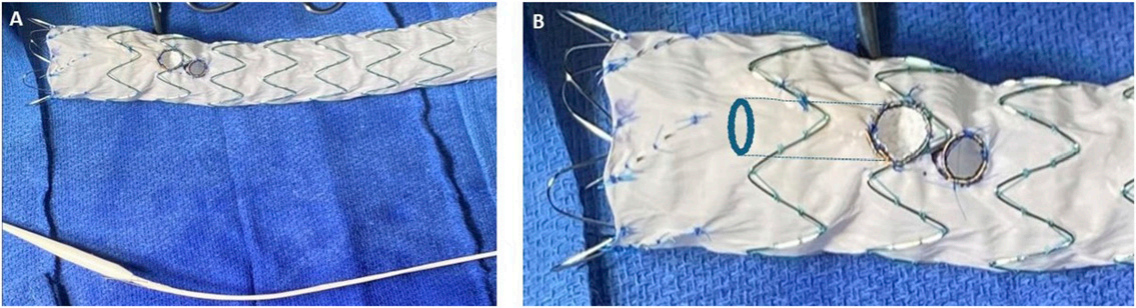
Cook Zenith Alpha proximal tapered graft used for procedure with fenestrations for the innominate and left carotid artery (A). Backtable modifications (B) were performed to create an internal branch at the innominate artery fenestration with a Viabahn stent.

**Figure 3.**
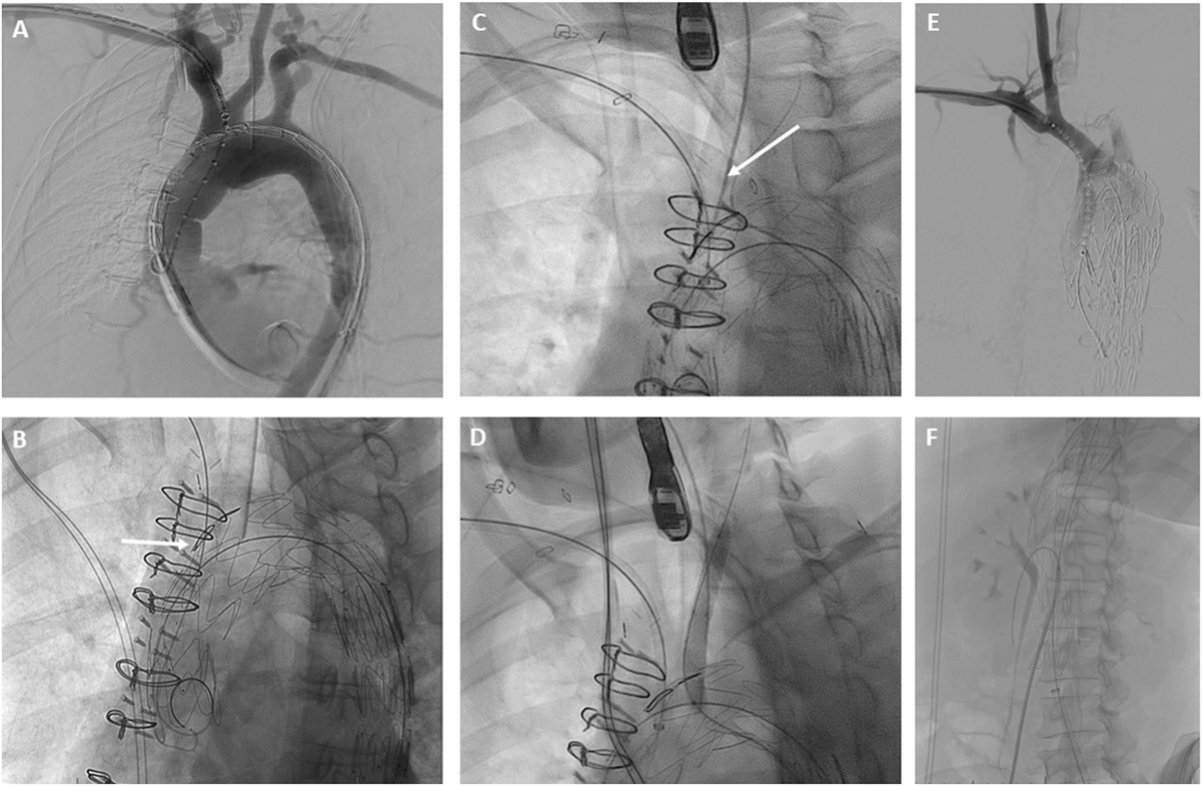
Operative angiogram sequence showing (A) Alpha Zenith endograft with modification for an internal branch for the innominate artery (IA) and fenestration for the left common carotid artery (LCCA). (B) IA fenestration cannulation (arrow). Left subclavian artery (LSCA) cannulation (C) and stenting (D). IA internal branch angiogram (E). Zenith dissection bare metal stent placement in the abdominal aorta (F).

While the anesthesiology team resuscitated the patient and prepared her for general anesthesia, a 34 mm × 209 mm Cook Alpha Zenith endograft (Cook Medical, Bloomington, IN) was modified adding an internal branch for the innominate artery (IA) and a fenestration for the left common carotid artery (LCCA). An antegrade internal branch was created for the IA using a 9 mm Gore Viabahn stent (W. L. Gore & Associates, Flagstaff, AZ) (Figure 2). The fenestration for the LCCA was reinforced with a goose neck snare wire. (Medtronic Santa Rosa, CA). To reduce the complexity of the case, the left subclavian artery (LSCA) was left to be covered with the endograft with proximal occlusion and embolization during the procedure. A 0.018-inch roadrunner wire (Cook Medical, Bloomington, IN) was used to secure the precurved cannula of the Zenith Alpha delivery system to the intended outer curve of the endograft. This spiral stabilizing wire, or “spine wire”, allows orientation of the endograft in the aortic arch with the branch/fenestration at the intended clock position along the outer curve. The device was re-sheathed back into the delivery system. Under general anesthesia, a proximal right brachial artery (BA) cutdown was performed and bilateral common femoral artery (CFA) percutaneous access was obtained. A .035” intravascular ultrasound (IVUS) catheter was introduced through the left CFA access to confirm access and placement of wires in the true lumen and to confirm registration of the live fusion imaging. The origins and corresponding positions of the IA, LCCA and LSCA on fluoroscopy were confirmed, and IV heparin was administered. Next, a Lunderquist wire (Cook Medical, Bloomington, IN) was positioned in the left ventricle and used for temporary rapid ventricular pacing with a grounding needle for PMEG deployment. The graft was positioned along the arch with its proximal end landing in the patient’s prior ascending aortic graft. The endograft was deployed under rapid pacing after an angiogram was performed to ensure proper positioning of the device (Figure 3A). The delivery system was then removed and exchanged for a distal extension 36 mm × 154 mm Cook Zenith TX2 endograft with overlap in the proximal DTA to obtain distal seal. Then, a Gore Trilobe (W. L. Gore & Associates, Flagstaff, AZ) balloon was advanced over the wire followed by post dilatation of the overlapping segment and distal seal zone. Selective cannulation of the IA branch was performed through the brachial access with a 0.035” glide wire, RBI catheter (Merit Medical, South Jordan, UT) and steerable sheath (Figure 3B). The wire was exchanged for a 035-in TAD2 wire (Abbott Vascular, Santa Clara, CA). The LCCA fenestration was selected through the left CFA access with a Bern catheter and a 0.035-inch Glide wire which was then exchanged for a 035-in TAD2 wire (Figure 3C). An 8 mm × 50 mm Viabahn was placed in the distal LCCA followed by an 8 mm × 59 mm VBX stent through the LCCA fenestration to obtain an optimal seal against the fenestration ring and flared with a 10 mm × 20 mm balloon (Figure 3D). Similarly, an 11 mm × 79 mm VBX stent was advanced and deployed in the IA into the inner branch and post dilated with a 14 mm × 40 mm Armada balloon (Figure 3E).

**Figure 4.**
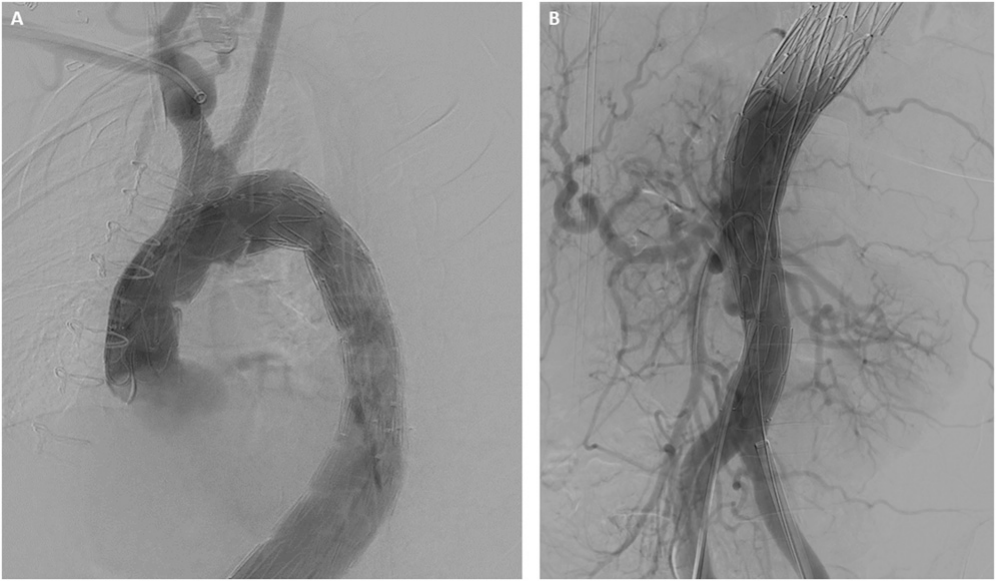
Completion arch (A) and abdominal aorta (B) angiogram with successful aneurysm exclusion and patency of target vessels.

**Figure 5.**
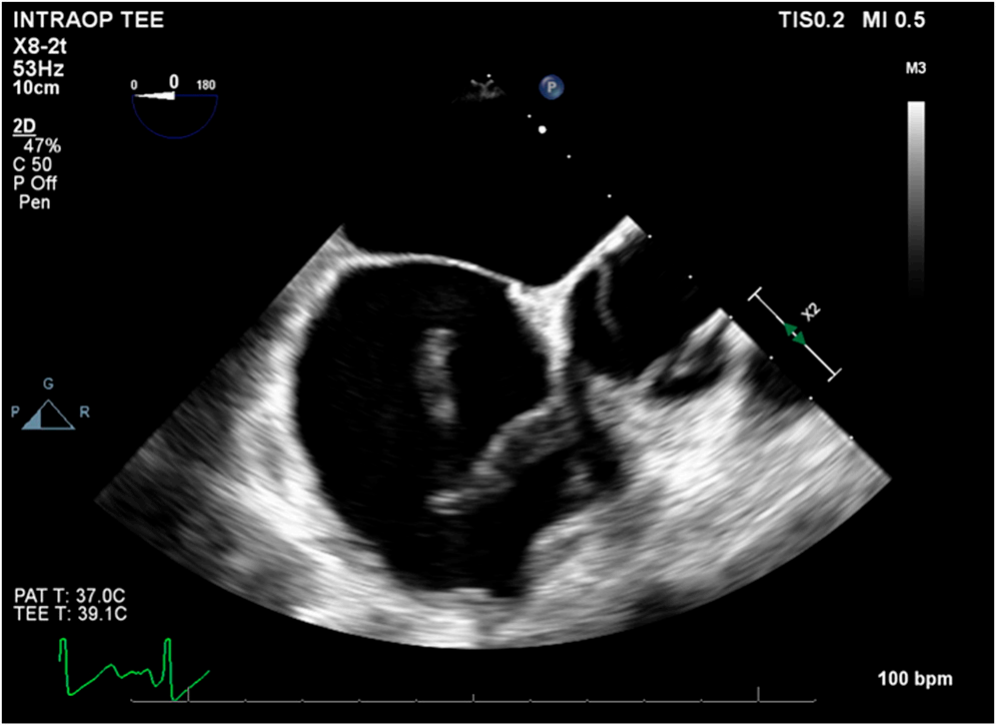
Intraoperative transesophageal echocardiogram with demonstration of atrial thrombus.

The IVUS was then used to interrogate the abdominal aorta and true lumen distal to the stent grafts, which demonstrated severe stenosis in comparison to the false lumen. A Zenith dissection stent (Cook Medical, Bloomington, Ind) was then deployed from the thoracic aorta to just proximal to the aortic bifurcation (Figure 3F). Completion angiogram demonstrated excellent endograft positioning and patency of the supraortic branches and visceral vessels (Figure 4). The proximal LSCA was embolized with a Microvascular Plug (Medtronic Santa Rosa, CA) and packed with coils via a left radial artery access. During intraoperative monitoring, a transesophageal echocardiogram revealed a mobile right atrial thrombus (Figure 5), so an inferior vena cava (IVC) filter was implanted in lieu of anticoagulation until complete seal could be confirmed on a postoperative CTA. After obtaining percutaneous right common femoral vein access with a 7 Fr sheath, IVC venography was performed. Next, a Gunther Tulip IVC filter (Cook Medical, Bloomington, IN) was deployed just below the renal veins, and adequate position was confirmed with completion venogram.

**Figure 6.**
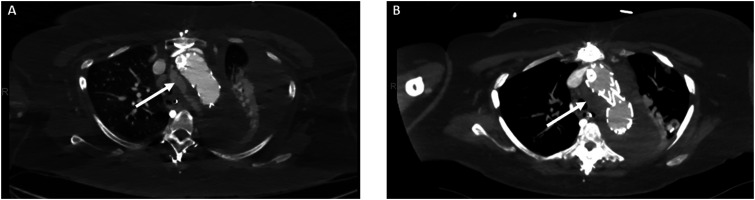
Postoperative day 1 CTA (A) with presence of type Ic endoleak (arrow) and adequate resolution after secondary intervention on postoperative day 5 (arrow) (B).

**Figure 7.**
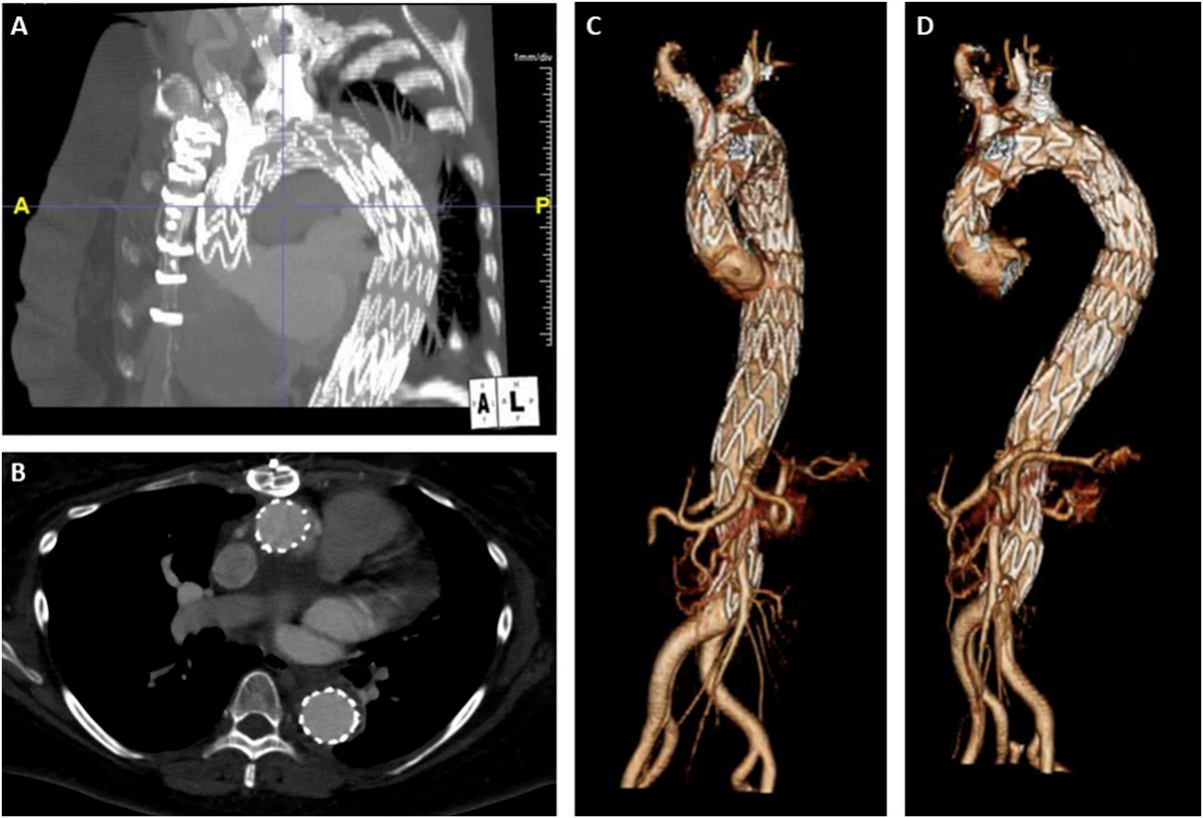
Sagittal (A) and axial (B) view of follow-up abdominal CTA with aneurysm sac shrinkage, PMEG vessel stent patency, and appropriate LSCA occlusion. Anterior (C) and anterolateral (D) 3D postoperative reconstruction of physician-modified endograft (PMEG) thoracic endovascular aortic repair (TEVAR).

Postoperatively, the patient was neurologically stable, transferred to the surgical ICU and planned for repeat CTA on postoperative day 1 prior to thoracostomy tube placement. On CTA imaging, we noted the presence of a type Ic endoleak at the IA branch (Figure 6A) and slight distal migration of the LSCA coils. She was taken back to the operating room for an extension of the IA stent, further LSCA coil embolization and thoracostomy tube insertion. BA cutdown was performed, followed by retrograde access into the IA stent. A Glidewire and glide catheter were advanced and the sheath was replaced by a 12 Fr Gore DrySeal sheath. After angiographic confirmation of the innominate bifurcation, a Gore Excluder 16 mm × 12 mm x 70 mm internal iliac limb was advanced and deployed just proximal to the right carotid artery takeoff. A Q50 balloon was employed for post dilation of the overlap zones with the previously placed IA stent and the iliac limb and completion angiogram demonstrated excellent seal without evidence of type 1B endoleak. For the migrated subclavian coil, we obtained percutaneous left BA access with a 4 Fr sheath and advanced a Rosen wire, which was also used to push the previous migrated coil pack back into the proximal LSCA. A Terumo AZUR CX 13 mm coil (Terumo Medical Corporation, Somerset, NJ) was then used to perform distal packing to the migrated coil and completion angiogram revealed excellent seal at the proximal LSCA with no evidence of endoleak into the aneurysm sac. On postoperative day 5, repeat CTA demonstrated resolution of the endoleak (Figure 6B), and she was started on anticoagulation for her PE. On POD 6, she was extubated and the thoracostomy tube was removed. IVC filter removal was performed on postoperative day 12, as she was doing well with anticoagulation. On postoperative day 14, the patient was discharged to home with family support. At 3-month OSH follow-up, the patient reported that she had returned to her baseline activities. 5-month and then 1 year follow-up CTA revealed aneurysmal sac regression, supraaortic vessel stent patency, and adequate LSCA occlusion (Figure 7). The patient will continue to have yearly monitoring with CT imaging.

## Discussion

The risks of TAA dissection and rupture increase in proportion to increased aneurysm size. Zafar et al studied the natural history of descending TAAs and thoracoabdominal aortic aneurysms (TAAA) most recently in 2021. Ninety-three percent of ruptures occurred for aneurysm diameter >5 cm, and a 19% yearly rate of dissection, rupture or death for aneurysm diameter >6 cm.^9^ For open surgical repair, permanent adverse outcomes (mortality, permanent neurologic event, or permanent renal failure necessitating hemodialysis at discharge) are independently associated to preoperative cardiac disease unrelated to the aorta, congestive heart failure, and current or remote smoking history.^10^ On the other hand, chronic aortic dissection, connective tissue disorders, and age ≤50 years are protective factors for postoperative adverse events.^11^ Recent evidence has reported 14% of thirty-day mortality for patients who undergo redo sternotomy.^12^ Thus, significant preoperative non aortic and aortic related comorbidities may have a significant impact on procedure success and outcomes, which must be taken into account for appropriate clinical decision for open vs endovascular repair.

In this case, we elected to proceed with branched-TEVAR due to critical hemodynamic compromise and high surgical risk for open repair. Lower morbidity and mortality as well as length of stay with TEVAR have changed current trends of intervention for descending TAA.^13,14^ Chiu et al. evaluated the effectiveness of TEVAR and open surgical repair (OSR) in the treatment of intact DTA aneurysms. While OSR had lower reintervention rates [(hazard ratio: 0.40 (95% CI: 0.34 to 0.60; *P* < 0.001)], mean survival time difference at 9 years was higher for TEVAR.^15^

PMEGs in TAAA have demonstrated feasibility for patients unfit for open repair.^16^ Their use in arch aneurysms is less well established though a few small series have reported good short-term results.^17,18^ In a study evaluating feasibility and early outcomes of 9 patients who underwent TEVAR with 3-vessel custom made devices (CMDs) and PMEGs, Lee et al. reported 100% of procedural technical success, and no in-hospital deaths for arch PMEGs. Overall, 66% of patients presented with endoleaks in the immediate postoperative period, with spontaneous resolution in 5 cases and reintervention for the LSCA for 1 patient. One case of late mortality was reported for PMEGs on postoperative day 142 due to endograft infection.^17^ Limited data exists for emergent endovascular arch repairs. Jędrzejczak et al reported a case of arch PMEG for a Zone 2 arch rupture after multi-organ trauma with parenchymal organ bleeding with successful outcomes for restoration of aortic arch continuity and appropriate inflow to the cerebral vessels.^19^ In the present case, given the need for emergent repair, we elected to make a 2-vessel endograft with an antegrade branch for the IA and a fenestration for the LCCA. LSCA embolization is usually well tolerated in emergency settings and the decision was made to limit the complexity of the case by avoiding a third branch or fenestration. We used an Intuition workstation (TeraRecon, San Mateo, CA) to determine the distances along the greater curve of the aorta from the proximal landing zone in the prior aortic graft to the origins of the IA and LCCA. Correct orientation of the graft in the aortic arch was achieved through a combination of the pre-curved cannula of the Cook Alpha endograft as well as by securing the curved cannula to the intended greater curve of the endograft by weaving a .018” wire in a spiral fashion along the length of the endograft and around the inner cannula. This “spiral wire” technique mimics that used in the Cook Medical Arch branch custom made device and is a key step to technical success. Rotational alignment in the aortic arch has to be achieved through self-alignment of the device rather than manipulation of the endograft as any torque applied to the device at the level of the femoral access to achieve alignment is unlikely to be transferred accurately to the endograft as it curves around the aortic arch. Additionally, excessive manipulation in the arch can increase the risk of stroke or retrograde dissection if the proximal landing zone is in native aorta.

Though technical success can be achieved in many of these cases with proper device design, long term outcomes with PMEGs in the arch still needs to be proven. Branch vessel stents in the arch face different forces than those in the thoracoabdominal aorta because of the dynamic nature of the ascending aorta and the arch. Additionally, limited data on the durability of current stent grafts in supra-aortic branch vessels exist. Nevertheless, early results of arch PMEG are encouraging, and long-term follow-up studies can bring further advances in device design to ensure durable repair. Our case represents the first report of a successful Zone 0 PMEG repair in the setting of a ruptured arch aneurysm.

## Conclusions

Endovascular management of ruptured aortic arch aneurysms with physician modified endografts is feasible. Adjunctive interventions may be required on a case-dependent basis. This approach can be an alternative for high surgical risk patients in emergent settings.
